# Weeklong improved colour contrasts sensitivity after single 670 nm exposures associated with enhanced mitochondrial function

**DOI:** 10.1038/s41598-021-02311-1

**Published:** 2021-11-24

**Authors:** Harpreet Shinhmar, Chris Hogg, Magella Neveu, Glen Jeffery

**Affiliations:** Institute of Ophthalmology, University College London, 11-43 Bath Street, London, EC1V9EL UK

**Keywords:** Neuroscience, Visual system

## Abstract

Mitochondrial decline in ageing robs cells of ATP. However, animal studies show that long wavelength exposure (650–900 nm) over weeks partially restores ATP and improves function. The likely mechanism is via long wavelengths reducing nanoscopic interfacial water viscosity around ATP rota pumps, improving their efficiency. Recently, repeated 670 nm exposures have been used on the aged human retina, which has high-energy demands and significant mitochondrial and functional decline, to improve vision. We show here that single 3 min 670 nm exposures, at much lower energies than previously used, are sufficient to significantly improve for 1 week cone mediated colour contrast thresholds (detection) in ageing populations (37–70 years) to levels associated with younger subjects. But light needs to be delivered at specific times. In environments with artificial lighting humans are rarely dark-adapted, hence cone function becomes critical. This intervention, demonstrated to improve aged mitochondrial function can be applied to enhance colour vision in old age.

## Introduction

Metabolic rate and ageing are both regulated by mitochondria. Mitochondrial membrane potential, however, declines with age resulting in reduced adenosine triphosphate (ATP) production, which is a major source of cellular energy. Cellular decline is further accelerated with ageing by increased production of pro-inflammatory reactive oxygen species (ROS)^1^. Retinal photoreceptors have the greatest mitochondrial density and metabolic demand in the body and age rapidly^2^. A pivotal point in their functional ageing in the human retina appears to be around 40 years^3^. Subsequently 30% of central rods progressively die, and while cones remain, they have reduced functionality^4–7^. Hence, regenerating aged cone function is vital because in an environment where artificial lighting results in humans rarely being fully dark-adapted and needing their rods, cone function becomes critical.

Exposure to long wavelength light (650–900 nm) in animals improves mitochondrial function, increasing ATP production and reducing ROS^8,9^. It also reduces the pace of age related cell death in the retina^10^. These mitochondrial changes translate into improved electrophysiological responses from the ageing retina in both insects and mice^11,12^. The mechanism may be due to reduced nanoscopic interfacial water layer viscosity around ATP rota pumps, increasing their efficiency^13^. As neuronal membrane pumps consume large amounts of ATP, mitochondrial decline impacts widely on the energy demands of the nervous system as age erodes pump efficiency^14^. Hence, improving ATP availability optically has potentially widespread impact including improved patterns of ageing in mammals and extended lifespan in insects^15^. While few human experiments have been undertaken with long wavelength light in ageing, daily exposures to relatively bright 670 nm light over weeks in aged humans has been demonstrated to improve rod and cone function^3,16^. But we have no knowledge of how much exposure is needed and how long its influence persists.

## Results

**Figure 1:**
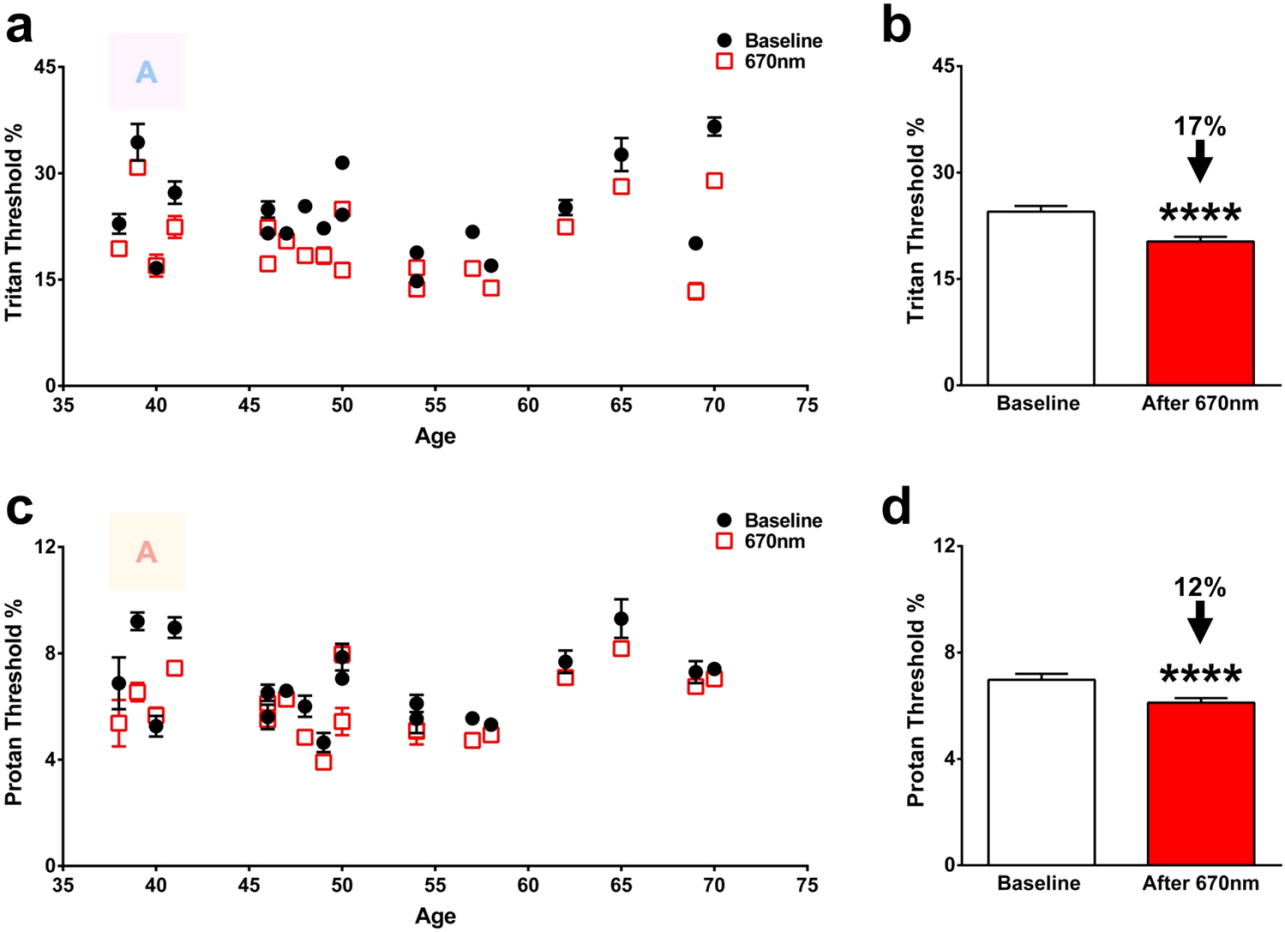
Colour contrast sensitivities (CCS) measured 3 h following a morning exposure (8-9AM) of 670 nm. CCS of tritan (**a**) and protan (**c**) axes measured in 20 healthy subjects and their response to 670 nm exposure. Black closed circles represent baseline measurements and red open boxes are those in the same individuals measured 3 h following a single 3 min 670 nm exposure delivered in the morning. The colour letter A in both graphs is an example of the target to be identified as it appeared on the screen. (**b**) Thresholds for tritan function in individuals 38–70 years. Most subjects displayed a significant decrease to their tritan thresholds following light exposure. Overall, there was a 17% reduction to thresholds across the population. (**d**) Thresholds for protan function in individuals 38–70 years, with half the population displaying a significant decrease to their protan thresholds after 670 nm exposure. Total protan thresholds were reduced by 12% across all subjects. Wilcoxon matched-pairs signed rank test was used for statistical analysis. Data are presented as means ± SEM. ****p < 0.0001. Age related differences were not apparent in the baseline measurements as was apparent in Shinhmar et al. (2020). This was because the age range covered here was shorter than in the previous study with a tendency for clustering in the middle age range.

A single 3 min 670 nm morning exposure to the eye at energy levels approximately a log unit greater than found in environmental light^17^ significantly improved subject colour contrast thresholds for both tritan and protan axes when tested 3 h later. Tritan threshold measures are improved by an average of 17% (P < 0.0001) and protan by an average of 12% (P < 0.0001. Figure 1). When the population is subdivided by progressive age, percentage threshold reductions for the tritan axis in those 38–49 years is 14% (p < 0.0001), in those 50–59 is 20% (P < 0.0001) and those over 60 is 19% (P < 0.0001). The same figures for the protan axis according to subdivided age are 13% (P < 0.001), 11% (P < 0.001) and 12% (P < 0.01) respectively.

**Figure 2:**
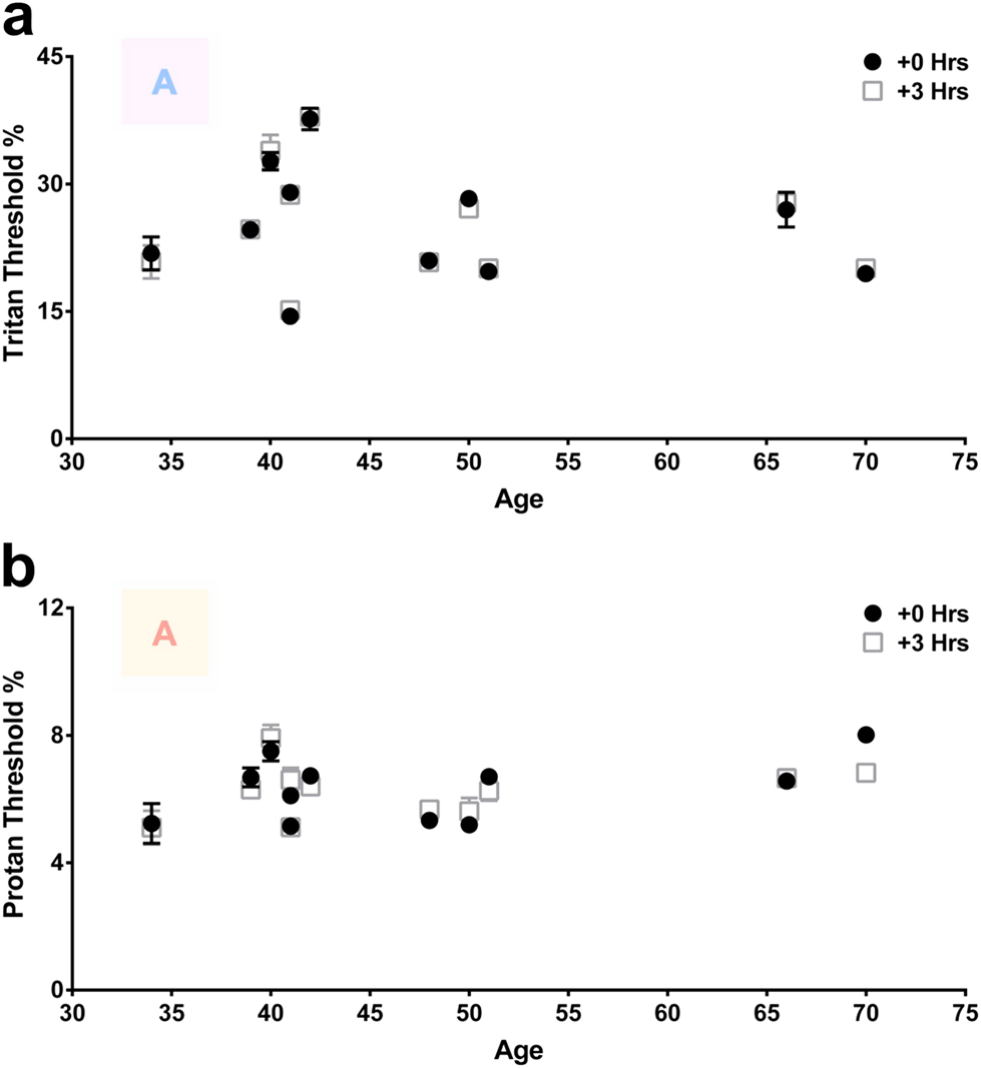
Colour contrast sensitivities (CCS) from a control cohort measured at the same time of day (8-9AM) with no 670 nm light exposure. CCS of tritan (**a**) and protan (**b**) axes measured in 10 subjects (34–70 years) at 8-9AM and 3 h later in the same manner as morning 670 nm light exposure and re-testing. Black closed circles represent measurements taken at 8-9AM to mimic baseline measures and time of 670 nm light exposure. Grey open boxes are those in the same individuals measured 3 h later between 11AM-12PM to mimic the time interval a perceived effect to 670 nm light exposure was achieved. The colour letter A in both graphs is an example of the target to be identified as it appeared on the screen. Thresholds for tritan function revealed no significant difference (p = 0.4648) when measured 3 h apart, simulating the same time of day for morning 670 nm light exposures. Similarly, thresholds for protan function revealed no significant difference (p = 0.9658) across all 10 subjects. Wilcoxon matched-pairs signed rank test was used for statistical analysis. Data are presented as means ± SEM.

Overall threshold reductions along the tritan axis were 17% for both males and females (P < 0.0001). Threshold reductions along the protan axis were 10% (P < 0.001) in males and 14% (P < 0.0001) in females. Subdividing by age group, visual axis and sex was not realistic because of the limited sample size. These changes are for measurements before and after 670 nm exposure within individuals. However, an additional control was undertaken where the subjects had their colour contrast thresholds measured at the same time in the morning as above and were then re-tested 3 h later without exposure to 670 nm light (Fig. 2). These showed no difference between the two measurements confirming that differences in the main group were due to 670 nm light exposure.

**Figure 3:**
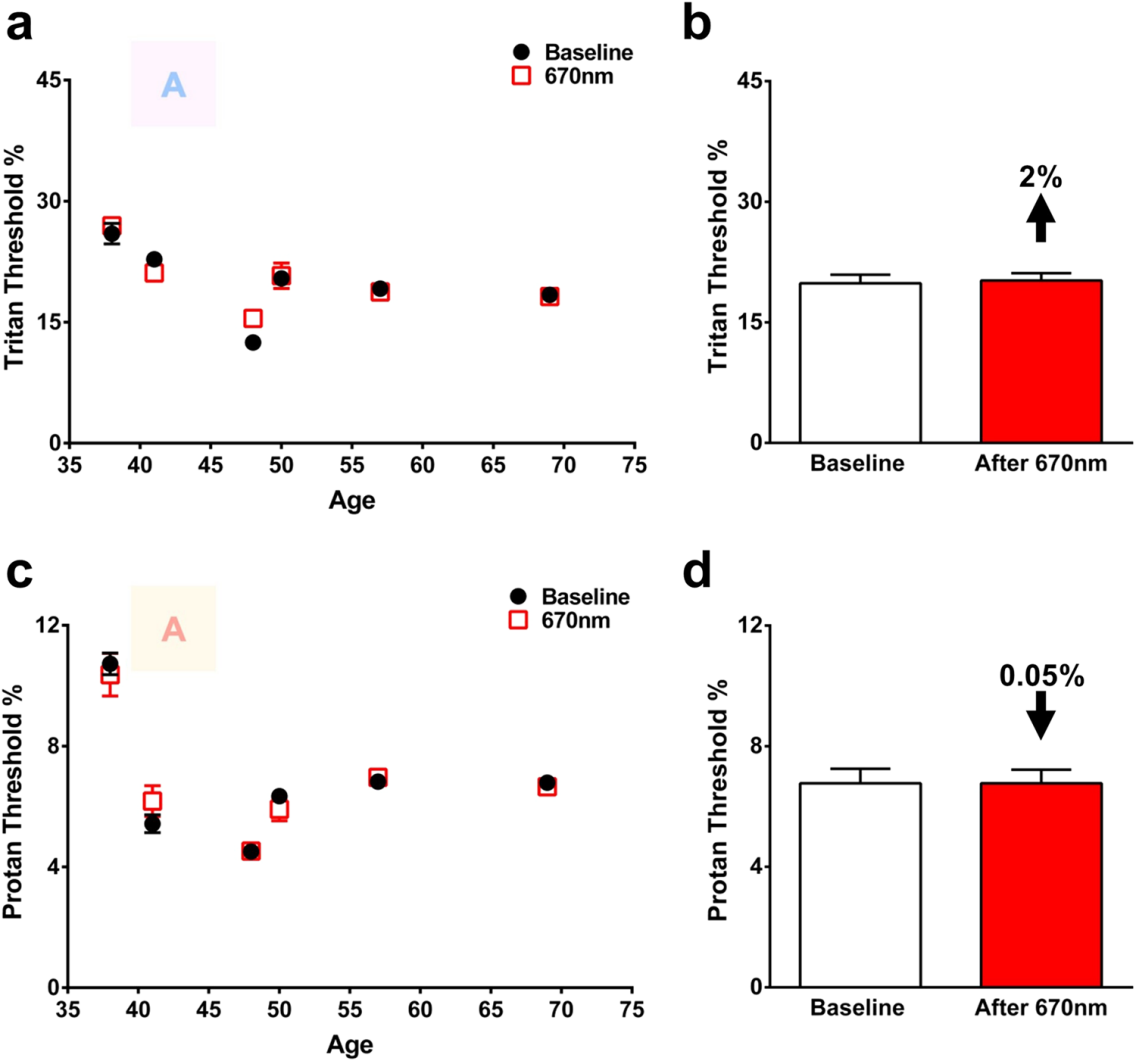
Colour contrast sensitivities measured 3 h following an afternoon exposure (12-1PM) of 670 nm. CCS of tritan (**a**) and protan (**c**) axes measured in 6 healthy subjects and their response to 670 nm exposure. Black closed circles represent baseline measurements and red open boxes are those in the same individuals measured 3 h following a single 3 min 670 nm exposure delivered in the afternoon. The colour letter A in both graphs is an example of the target to be identified as it appeared on the screen. (**b**) Thresholds for tritan function in individuals 38–69 years. No significant change to tritan thresholds (p = 0.3047) were found when subjects were exposed to 670 nm light in the afternoon. (**d**) Thresholds for protan function in individuals 38–69 years. 670 nm exposure showed no effect on protan thresholds (p = 0.2577) when it was delivered in the afternoon. Wilcoxon matched-pairs signed rank test was used for statistical analysis. Data are presented as means ± SEM.

**Figure 4:**
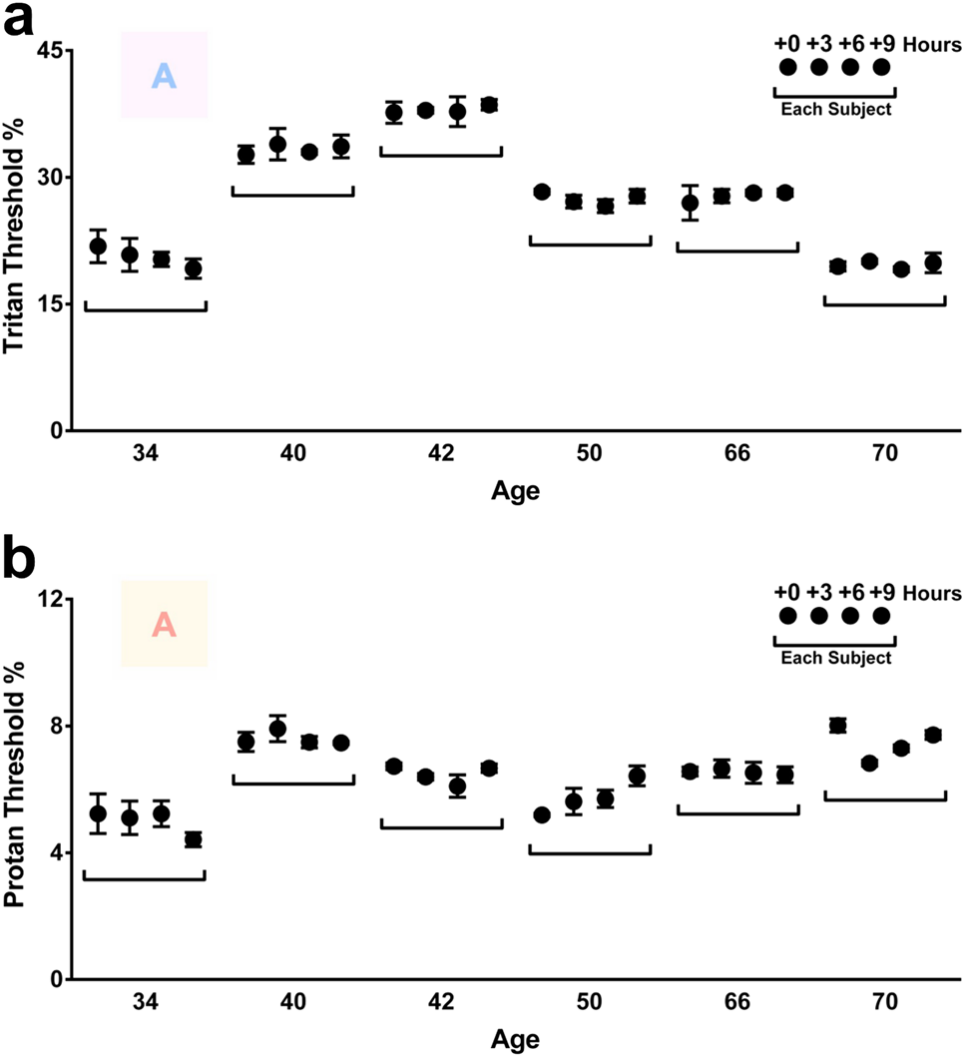
Variation in colour contrast sensitivities (CCS) across the day in 6 subjects. CCS of tritan (**a**) and protan (**b**) axes measured in 6 subjects (34–70 years) in 3 h intervals across the day. The four black closed circles within the bar represent each subject, measurements were taken in 3 h intervals at 8AM, 11AM, 2PM and 5PM. The colour letter A in both graphs is an example of the target to be identified as it appeared on the screen. Thresholds for tritan function revealed no significant variation across the day when measured in a 34 (p = 0.6898), 40 (p = 0.8902), 42 (p = 0.9388), 50 (p = 0.3890), 66 (p = 0.8592), and 70 (p = 0.7613) year-old. Similarly, thresholds for protan function revealed no significant variation across the day when measured in a 34 (p = 0.5802), 40 (p = 0.6149), 42 (p = 0.1937), 50 (p = 0.0943), 66 (0.9588), and 70 (p = 0.2222) year-old. An ordinary 1-way ANOVA and a multiple comparison test was used for inter-subject comparison and statistical analysis. Data are presented as means ± SEM.

Similar methods were applied to a subgroup (N = 6, Fig. 3) of subjects across a comparable age range with exposure to 670 nm light in the afternoon and tested 3 h later. The results showed no impact across either colour axis following afternoon light exposure. Hence only the morning light dose effective. However, it is possible that there are fundamental differences in the patterns of colour contrast sensitivities over the day that could explain this finding independent of 670 nm light exposure. Consequently, a subgroup of individuals covering the full age range, had their colour contrast thresholds measured repeatedly across the day at time 0, + 3, + 6 and + 9 hs. Data for protan measurements were more variable within subjects than tritan, but neither showed any consistent pattern between across time in the subjects (Fig. 4).

**Figure 5:**
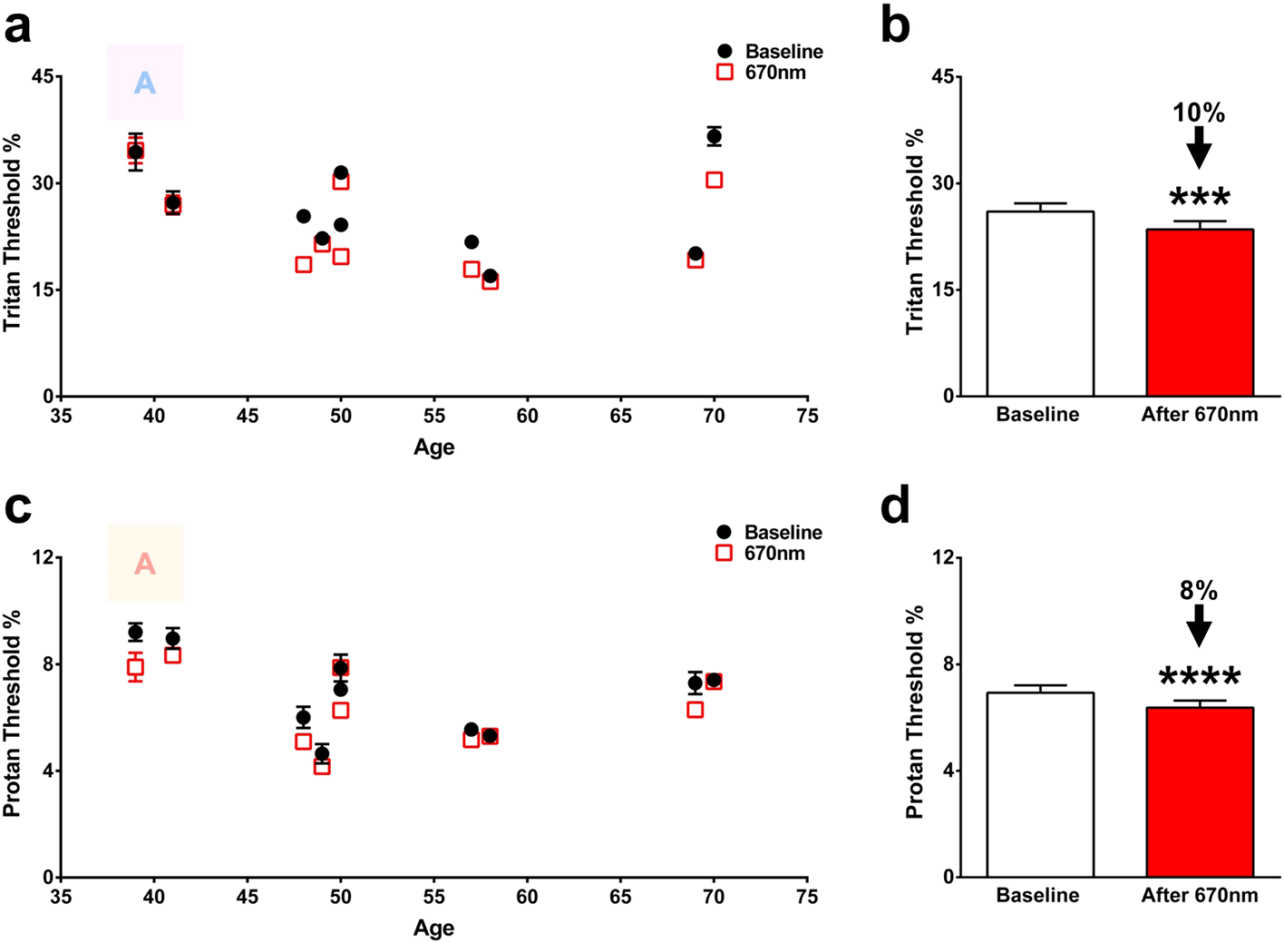
Colour contrast sensitivities measured 1 week later from a morning exposure of 670 nm. CCS of tritan (**a**) and protan (**c**) axes from 10 healthy subjects with their response to a single dose of 670 nm measured 1 week later, without any further 670 nm exposure in the intermediary period Black closed circles represent baseline measurements and red open boxes are those in the same individuals measured 1 week later from a single 3 min 670 nm light exposure delivered in the morning. Some subjects displayed a return of their thresholds to baseline, whereas some still had a reduction. The colour letter A in both graphs is an example of the target to be identified as it appeared on the screen. (**b**) Thresholds for tritan function in individuals 39–70 years. There was a 10% sustained reduction to tritan thresholds (p = 0.0001) across the population from baseline measures. (**d**) Thresholds for protan function in individuals 39–70 years. There was an 8% sustained reduction to protan thresholds (p < 0.0001) across the population from baseline measures a week following 670 nm exposure. Wilcoxon matched-pairs signed rank test was used for statistical analysis. Data are presented as means ± SEM. ***p ≤ 0.0001, ****p < 0.0001.

A subgroup (N = 10, Fig. 5) of subjects covering a comparable age range that had light exposure in the morning were re-tested 1 week later, without any further 670 nm exposure in the intermediary period. Significant threshold improvements in this population remained across both colour axes, although reduced compared to the 3 h measurements. For the tritan axis, threshold reductions were 10% (P < 0.001) and for the protan axis, they were 8% (P < 0.0001). Subdividing this population further was not feasible because subject numbers were not large enough.

## Discussion

Single exposures to 670 nm light delivered in the morning, at only 8 mW/cm^2^ have the ability to improve cone photoreceptor function in aged subjects to levels commonly found in much younger individuals and can be sustained for up to a week^3^. This result is substantiated in both a within subject repeated measure design and also compared to a control group which received no light. Our subject population was ≥ 35 years because we have previously shown that the impact of 670 nm light on retinal function is reduced in younger subjects, presumably due to the relatively healthy state of their retinal mitochondria^3^. This is consistent with the mitochondrial theory of ageing^1^.

Time of exposure is critical, as 670 nm light is only effective in the morning. This time dependent effect is likely due to the demonstrated shift in mitochondrial function across the day, and light exposure is likely only effective when synchronised to an aspect of this process. The mitochondrial biology behind shift over the day includes variation in complex activity and ATP production. Here the differential impact of 670 nm has been mapped in fly^18^. Within the mornings when mitochondrial function can be improved, there are times when mitochondria appear to be particularly sensitive. However, it would be surprising if their function could be improved uniformly over this period. Hence, we are currently mapping sensitivities over this time.

Despite the clarity of our results, some of the data are noisy. While positive effects are clear for individuals following 670 nm exposure, the magnitude of improvements can vary markedly between those of similar ages. Hence, caution is needed in interpretation of our data. It is possible that there are other variables between individuals that influence the degree of improvement that we have not identified so far and would require a larger sample size.

Previous studies in animals and on the human retina have used much higher energies than those applied here, which are approximately a log unit higher than found in environmental light^17^. A common target is approximately 40 mW/cm^2^ ^3,19^. But the energies used here are significantly lower at only 8 mW/cm^2^. Hence, both the number of exposures and the energy applied is much lower than previously reported.

Tritan function was more responsive to 670 nm light exposure than protan, and this difference likely relates to the respective mitochondrial populations of different cone types. Short wavelength sensitive cones uniquely have relatively few mitochondria and appear to derive their ATP via glycolysis^20^. They are also selectively vulnerable in ageing and metabolic diseases^21^. Mitochondria absorb strongly at 420 nm^22^ and their presence in the light path of S cones would reduce photons reaching the outer segments of these. This may be the reason why S cones have switched to glycolysis to produce ATP^20^. It is possible that 670 nm exposure results in the recruitment of what may be otherwise relatively few dormant mitochondria in S cones in terms of ATP production. If correct, then it is possible that the signatory characteristics of S cones including early saturation may change due to increased ATP availability^23^.

Our choice of measuring cone function at 3 h was based on our finding that this was the time needed to increase ATP in experiments on aged flies exposed to 670 nm^24^, but improved whole body respiration occurs in less than 0.5 h post 670 nm exposure in the fly^24^. The difference in timing may be due to shifts in patterns of ATP production versus its consumption. However, there is a need to determine the temporal course of increasing improvements in human cone function following 670 nm exposure. It is likely that this may start much earlier than shown in this study. Despite this, it is clear that improved colour contrast remains for both colour domains for up to a week. Here there is more consistency between fly and human data, as improved respiration in aged flies following 670 nm exposure can be found to be sustained for over 4 days^24^.

We have employed a stringent colour contrast test, which is used in many clinical environments for a range of colour defects^25–29^ but is not otherwise available widely. We are currently exploring if the improvements we find with 670 nm can also be detected using the more labour intensive, but more accessible Farnsworth Munsell 100 Hue test. However, as a measure of sensitivity, subjects were asked after 670 nm exposure if they thought their colour perception had changed in their normal visual environment and around 10% reported a subjective difference.

This study cannot rule out the possibility of adaptive mechanisms to the 670 nm exposure, although this is unlikely. But in data not reported in our previous study where 670 nm exposure was delivered daily for 2 weeks^3^, we did examine interocular transfer. There was no difference found in the unexposed eye over the treatment period. Hence, the repeat measures design for testing which here shows the mechanism found with a single exposure is almost certainly retinal.

The energies at 670 nm that we have used are within a log unit of those that can be found in sunlight. This naturally raises the question of whether daylight may impact positively on mitochondrial function in the retina. While this may be the case, we are only recently coming to appreciate the optical absorbance characteristics of mitochondria and their functional consequences. Mitochondria strongly absorb at shorter wavelengths^22^ that are present in environmental light and these are known to reduce their function^30–32^. Consequently, long, and short wavelengths in sunlight may have opposing and perhaps balancing influences on mitochondrial function in the retina.

Humans live in a world where we now rarely employ rod function exclusively because we control our light environment. This in combination with the fact that unlike rods, ageing cones do not die but have reduced function highlights the critical need to improve cone ability in progressively ageing populations. We demonstrate that we can significantly improve cone mediated colour contrast thresholds for a week using a single 3 min 670 nm light exposure by an average of 17% and in some older subjects by > 20%. This simple and highly economic intervention applied at the population level will significantly impact on the quality of life in the elderly and likely result in reduced social costs that arise from problems associated with reduced vision.

## Methods

The study was conducted in accordance with the Declaration of Helsinki and approved by the UCL research ethics committee (16547/001). Each participant provided written informed consent prior to testing.

Subjects were healthy, of both sexes with an age range of 34–70 years. Each had normal colour vision and were given a general questionnaire regarding eye health prior to testing. Different numbers of subjects were used in different experiments but all experiments on each were tested for thresholds of colour contrast sensitivity along both tritan and protan axes. Anonymised subject identity numbers, their ages and cohort of the experiment in which they were used are given in Table 1. In the case of multiple testing per subject, they were given a wash out period to the effects of 670 nm light for a few months. All exposures to 670 nm were for 3 min to the subjects’ dominant eye either in the morning between 8 and 9 AM or the afternoon between 12 and 1 PM and were assessed at either 3 h post exposure or 1 week later without further 670 nm exposure in the intervening period. Morning exposures measured 3 h later comprised 20 subjects (13 Females and 7 Males). Afternoon exposures measured 3 h later comprised of six subjects (three Females and three Males). For morning exposures measured 1 week later there were ten subjects (seven Females and three Males). The above were a repeat measures design within subjects. However, an additional control was undertaken where colour contrast thresholds were measured in the morning and then re-measured 3 h later without exposure to 670 nm. This comprised of ten subjects (six Females and four males). To determine if there were significant shifts in colour contrast sensitivities across the day that were independent of 670 nm and might undermine outcome measures for their exposure, six subjects were repeatedly tested at 0, + 3, + 6 and + 9 h (four Females and two Males).

**Table 1:** Subject demographics: a breakdown of the subjects used in different parts of the study.

	Age	Gender	Cohort	Subject also in Shinhmar et al*.* 2020
1	34	F	d, e	Yes
2	38	F	a, d	
3	39	F	a, b, c, d, e	
4	40	F	a, d	Yes
5	41	M	a, b, c, d	
6	42	F	d, e	Yes
7	46	F	a	
8	46	M	a	
9	47	M	a, d	
10	48	F	a, c	
11	49	F	a, b, c, d, e	Yes
12	50	F	a, c	Yes
13	50	F	a, b, c, d	
14	54	M	a	
15	54	F	a	
16	57	F	a, c	
17	57	M	b	
18	58	F	a, c	
19	62	F	a	
20	62	F	a	
21	65	M	a	
22	66	M	d, e	Yes
23	69	M	a, b, c, d, e	Yes
24	70	M	a, c	

*F* Female, *M* Male.

a = AM light exposure.

b = PM light exposure.

c = AM light exposure tested a week later.

d = AM control, no light exposure.

e = variation across the day.

The 670 nm light devices were supplied by CH electronics (UK) and based on commercial DC torches with nine 670 nm LEDs mounted behind a light diffuser so that energies at the cornea were approximately 8 mW/cm^2^. 670 nm light was delivered down a white internally reflecting tube that fitted over the eye with an internal diameter of 3.2 cm. Based on subject’s perception the region of the retina illuminated was centred on the macular and extended into the equator but did not include the far periphery. Estimates of the exact retinal region of illumination are hard to derive because the pupil will variably close in response to the light. However, 670 nm will penetrate the iris^33^ and this will most likely be associated with scatter. The energy delivered at this wavelength is less than a log unit greater than that found in environmental light^17^.

Colour contrast sensitivity (CCS) was measured based on the ChromaTest and a computer graphics system called Cavity, which assesses colour contrast thresholds across the protan axis (red-green visual axis,) and tritan axis (blue-yellow visual axis) axes^25–29^. Table 2 below defines the coordinates of these axes in the CIE colour space. These coordinates are the endpoints and are not adjusted for any subject according to the flicker balance.

**Table 2:** End-point x, y coordinates defining the protan and tritan axis according to the CIE colour space.

	x	y	x	y
	Red		Green	
**Protan**	0.494	0.386	0.336	0.475
	Blue		Yellow	
**Tritan**	0.299	0.23	0.433	0.439

Subjects were seated at a fixed distance from the stimulus monitor so that that opto-type letter subtended a 1.3-degree angle on the retina. Target letters were displayed against a background of the opposing colour with the colour contrast between the 2 varying and appeared either in red or blue for the protan and tritan axes respectively. The subject discrimination task was to identify the presented letter at varied contrasts against the background. Contrast was defined as 0% when the letter had the same hue as the background and 100% when the letter and the background difference was at its maximum achievable by the monitor. Hence, for example, a low contrast letter T presented in red on a blue background where target and background were isoluminant. Isoluminace was set for each subject prior to threshold testing, by making a heterochromatic flicker balance match between red and green and blue and green monitor guns until there is minimum flicker i.e. a balanced output for each subject taking into account the subject’s optical pathway. The output is used to adjust the values in the look-up table to produce equiluminous colours for the subject being tested. Thresholds were then determined by a modified binary search algorithm where if a response is correct, on the next presentation the colour difference between letter and background is halved. If the response is incorrect, the colour contrast is increased by half. This method of determining thresholds leads to finite steps which reach a plateau at the subject’s threshold, the minimum colour contrast required to identify a letter correctly.

We used a within subjects repeat measures design because this reduces effects due to individual differences independent of the metric examined. Further, separate controls using different wavelengths of light could not be used because a wide range of wavelengths impact on mitochondrial function both positively and negatively and these are present in different proportions in white light^31,32^. All results were taken in triplicate and analysed from initial baseline recordings and final recordings taken after 670 nm exposure at the indicated time points. Test–retest variability was 1.6% for protan thresholds and 6% for tritan thresholds (N = 20 subjects) calculated from separate normative data with identical equipment and methods. The test–retest variability was calculated via a repeatability coefficient as 1.96√2σ. Data were graphed and analysed using GraphPad Prism 6 (GraphPad, San Diego, CA) with a Wilcoxon matched-pairs signed rank test for significance, and an ordinary 1-way ANOVA for inter-subject comparison in variation across the day.

## Data Availability

The data that support the findings of this study are available on request from the corresponding author GJ. The data are not publicly available due to them containing information that could compromise research participant privacy.
